# A High-Level Fungal Diversity in the Intertidal Sediment of Chinese Seas Presents the Spatial Variation of Community Composition

**DOI:** 10.3389/fmicb.2016.02098

**Published:** 2016-12-23

**Authors:** Wei Li, Mengmeng Wang, Xiaomeng Bian, Jiajia Guo, Lei Cai

**Affiliations:** ^1^College of Marine Life Sciences, Ocean University of ChinaQingdao, China; ^2^State Key Laboratory of Mycology, Institute of Microbiology, Chinese Academy of Sciences (CAS), BeijingChina

**Keywords:** microbiome, metabarcoding, fungal community, distribution pattern, intertidal region

## Abstract

The intertidal region is one of the most dynamic environments in the biosphere, which potentially supports vast biodiversity. Fungi have been found to play important roles in marine ecosystems, e.g., as parasites or symbionts of plants and animals, and as decomposers of organic materials. The fungal diversity in intertidal region, however, remains poorly understood. In this study, sediment samples from various intertidal habitats of Chinese seas were collected and investigated for determination of fungal community and spatial distribution. Through ribosomal RNA internal transcribed spacer-2 (ITS2) metabarcoding, a high-level fungal diversity was revealed, as represented by 6,013 OTUs that spanned six phyla, 23 classes, 84 orders and 526 genera. The presence of typical decomposers (e.g., *Corollospora* in Ascomycota and *Lepiota* in Basidiomycota) and pathogens (e.g., *Olpidium* in Chytriomycota, *Actinomucor* in Zygomycota and unidentified Rozellomycota spp.), and even mycorrhizal fungi (e.g., *Glomus* in Glomeromycota) indicated a complicated origin of intertidal fungi. Interestingly, a small proportion of sequences were classified to obligate marine fungi (e.g., *Corollospora, Lignincola, Remispora, Sigmoidea*). Our data also showed that the East China Sea significantly differed from other regions in terms of species richness and community composition, indicating a profound effect of the huge discharge of the Yangtze River. No significant difference in fungal communities was detected, however, among habitat types (i.e., aquaculture, dock, plant, river mouth and tourism). These observations raise further questions on adaptation of these members to environments and the ecological functions they probably perform.

## Introduction

The intertidal region is the area rhythmically covered by water or exposed to air, which is highly influenced by anthropogenic activities and climate changes (Raffaelli and Hawkins, 2012). The dynamic environment with extreme shifts in moisture, waves and salinity may be stressful for some organisms living in this region. On the other hand, the intertidal region provides a broad range of habitats such as mangrove, seagrass meadow, sandy beach, rocky shore, shallow coral and aquaculture area (Nordlund et al., 2014). Recent studies suggest that the intertidal region probably supports a vast biodiversity of microorganisms (Azovsky et al., 2013; Boehm et al., 2014). For example, using 454-pyrosequencing, thousands of unique bacterial OTU had been identified from intertidal sands of the California coast (Boehm et al., 2014). Among microorganisms, fungi play important roles in the process of decomposition and mineralization of organic matter in marine ecosystems (Hyde et al., 1998; Jones, 2011; Velez et al., 2013). However, only a few fungal species have been identified and detected from intertidal habitats such as mangrove, wood substrate and alga, through traditional isolation approach (Jones et al., 2006; Suryanarayanan et al., 2010; Pang et al., 2011; Kachalkin, 2014; Rämä et al., 2014; etc.). Next-generation sequencing-based studies are rare, except Arfi et al. (2012) who investigated the anoxic mangrove sediments of Saint Vincent Bay, and revealed that Agaricomycetes was the dominant fungal class. As a result, insufficient information on the fungal community in the sediment of intertidal ecosystems is hindering our understanding of their ecological functions (González et al., 1998; Sallenave-Namont et al., 2000; Rämä et al., 2014).

China is one of the largest coastal countries and includes four marginal seas, namely the Bohai Sea (BHS), Yellow Sea (YS), East China Sea (ES) and South China Sea (SS) (Liu, 2013). The intertidal region along the Chinese coastal seas consists of various types of ecosystems (e.g., sand beach, cultured mud flat, and mangrove), all of which provide important habitats for marine organisms. Traditional survey on fungal diversity using culture dependent methods have recorded a few species mostly from mangrove of Taiwan province (Pang et al., 2011), marine sponges and algae (Zhou et al., 2011; Cheng et al., 2015), and woody substrates collected from the intertidal region of Hong Kong (e.g., Vrijmoed et al., 1986, 1994). No investigation has been reported using molecular tools for determination of intertidal fungal community. Additionally, owing to the economic development, the coastal ecosystem in China is clearly degraded, which results in the biodiversity decline (Liu, 2013; He et al., 2014). Therefore, investigation of fungal diversity will provide essential information for the conservation of biodiversity, as well as bio-resources utilization.

The objectives of the current study were: (1) to estimate the fungal diversity and the ecological roles they probably perform in the Chinese intertidal region; and (2) to reveal the differences in community composition among sea regions (i.e., the BHS, YS, ES, and SS) and habitat types (i.e., aquaculture, dock, plant, river mouth and tourism). To achieve our goals, 43 sediment samples collected from the Chinese intertidal zone spanning about 12,000 km of mainland coastline were investigated for fungal diversity through DNA metabarcoding.

## Materials and Methods

### Sediment Sample Collection

**FIGURE 1:**
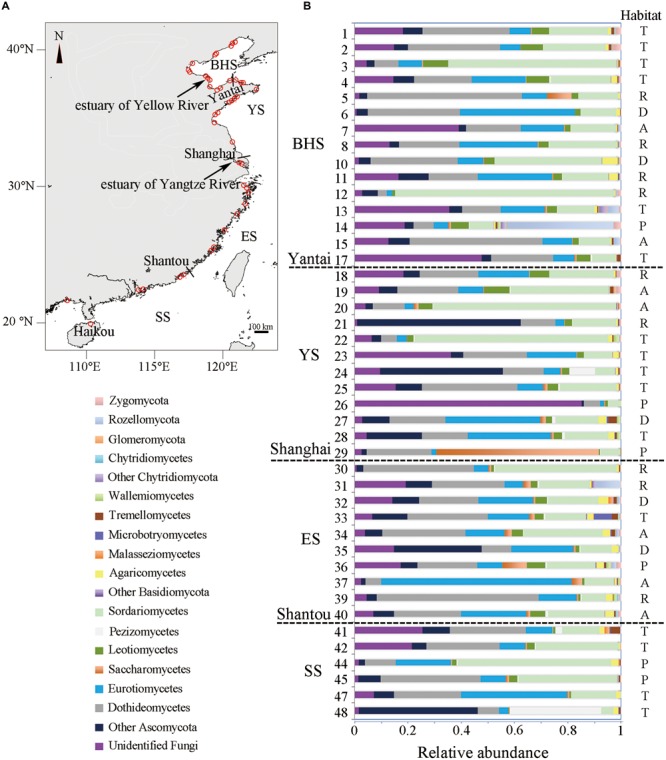
**(A)** Location of 43 sampling sites along the Chinese intertidal zone (detailed information and α-diversity of each site are listed in **Supplementary Table S1**). The short lines show the relative locations of three Chinese cities. **(B)** Relative abundance of 19 main taxonomic groups in each site. Habitat type of each site is shown (A, aquaculture; D, dock; P, plant; R, river mouth; and T, tourism). The legend in the lower left gives the 19 main taxonomic groups that are in order in accordance with the sequence of relative abundance shown in each site from left to right.

On the basis of environmental characteristics, intertidal region can be recognized as four zones that include infrabeach, mesobeach, suprabeach and the terrestrial domain (González et al., 1998). Among them, mesobeach is the area which is continually covered by water and rhythmically exposed to air. Moreover, the major quantity of organic remains is accumulated in the mesobeach area. Therefore, during low tide in June 2014, 43 sediment samples were collected from typical mesobeach of the Chinese seas, which include the BHS (15 samples), YS (12 samples), ES (10 samples) and SS (6 samples) (**Figure 1A**, **Supplementary Table S1**). Each sample contained five subsamples that were collected from one sampled site of the intertidal zone. Each sampled site was an approximately rectangular area of about 50 m by 5 m, where five subsamples were collected randomly. Then the five subsamples mixed thoroughly as a sample. Sediments were collected from sampled sites using 3.5 cm-inner-diameter pre-cleaned glass tubes sealed with sterile plastic film. These glass tubes filled with sediment were transported to the laboratory and immediately stored at -80°C until total DNA extraction was performed. For analysis of the distribution patterns of fungal communities across different habitat types, the sampled sites were designated as one of five habitat types, namely the area of aquaculture, dock, plant (where covered with mangroves and reeds), river mouth or tourism, on the basis of the main function of a specific site (Nordlund et al., 2014; Whitfield and Pattrick, 2015).

### DNA Extraction and HiSeq-Sequencing

In the laboratory, aliquots of sediment (2–8 cm in depth) from the central parts of the cores of each subsample were removed using a sterile spatula and mixed thoroughly. Genomic DNA extraction was performed on 0.5 g of composite sediment using the FastDNA^®^ Spin kit for soil (MP Biomedicals, Solon, OH, USA) according to the manufacturer’s instructions.

The ITS2 rDNA region was amplified using the fungal-specific primer pair ITS3/ITS4 (White et al., 1990). A reverse or forward primer was supplemented with one of the 86 5-base identifier barcodes (**Supplementary Table S2**). PCR was carried out in five replicates using the following thermocycling conditions: an initial hot start incubation (5 min at 94°C) followed by 34 cycles of denaturation at 94°C for 30 s, annealing at 55°C for 30 s and extension at 72°C for 1 min, followed by a final extension at 72°C for 15 min. PCR products were pooled and their relative quantity was estimated by running 5 μL amplicon DNA on 2% agarose gel, and products were purified with GeneJET^TM^ Gel Extraction Kit (Thermo Scientific). Amplicon libraries were generated using NEB Next^®^ Ultra^TM^ DNA Library Prep Kit for Illumina (NEB, USA) following the manufacturer’s recommendations and index codes were added. Samples were sequenced using the Illumina HiSeq 2500 sequencing platform. The HiSeq fastq files and the identifier barcode files were deposited in the National Center for Biotechnology Information Sequence Read Archives (SRA) as BioProject ID PRJNA329389.

### Bioinformatic Analysis

Raw sequences were paired using FLASH (Magoč and Salzberg, 2011), quality filtered and assigned to samples based on the identifier barcodes using the QIIME pipeline (Caporaso et al., 2010) with the following options: -*q* = 19, -*r* = 3, and -*p* = 0.75. Then, ITSx 1.0.11 (Bengtsson-Palme et al., 2013) was applied to trim out 5.8S rRNA and 28S genes and remove the compromised and non-target sequences with setting minlength for ITS2 of 99 bases and other parameters as default (Tedersoo et al., 2014). Uchime_ref command in USEARCH (Edgar, 2013) was used to detect and remove chimeras using the unified system for DNA based fungal species (UNITE) and international nucleotide sequence databases (INSDC) fungal ITS databases (version released on Jan. 31, 2016) as reference (Kõljalg et al., 2013; Nilsson et al., 2015). These filtered sequences were clustered to operational taxonomic units (OTUs) at 97% sequence similarity threshold with cd-hit 4.6.1 (Fu et al., 2012). Singletons were removed, as they tend to be artifactual (Tedersoo et al., 2010; Lindahl et al., 2013).

The longest sequence of each OTU was selected as the representative (**Supplementary Table S7**) for local BLASTn 2.2.30+ search (Altschul et al., 1997) with a conservative setting (word_size = 7, penalty = -3, reward = 1) against UNITE + INSDc database. Rules based on BLASTn *E*-value and sequence similarity thresholds were employed for robust identification (Tedersoo et al., 2015). The BLASTn *E*-values < e^-50^ and sequence similarity > 75%, over 70% sequence length allowed robust assignment to the fungal kingdom. However, query sequences with an *E*-value between e^-20^ and e^-50^ were manually checked against the 100 best-matching sequences for accurate assignment. These above queries were assigned to the fungal kingdom and were labeled by genus, family, order, or class at 95, 90, 85, and 80% sequence identity, respectively. For the classes Eurotiomycetes, Leotiomycetes, Sordariomycetes, and order Cantharellales, which contain multiple lineages appeared in this study, a decline of 5% identity was applied. The *E*-value > e^-20^ was used as threshold to classify sequences as “unknown.”

For further robust assignment, NAÏVE BAYESIAN CLASSIFIER (NBC) with the Warcup ITS training set as reference (Wang et al., 2007; Deshpande et al., 2016) was performed. The NBC-based taxonomic assignment is considered to be credible only when node bootstrap support is above 50%. In addition, we attempted to use a criterion for classification of OTUs to the level of species. A representative sequence (RS), which has the values of 100% identity and 100% coverage against a reference sequence from BLASTn, and a 100% node bootstrap support from NBC concurrently, could be assigned to the level of species.

### Statistical Analyses

The relative abundance of an individual taxon (e.g., class) within each sample was estimated by comparing the number of reads assigned to each taxon for individual samples. For comparative analysis of α-diversity (OTU richness, Shannon index and evenness value) across different sites sampled, the data set without singletons was rarefied to 12,404 reads (the lowest number of reads recovered for our 43 samples) using the vegan package of R (R Development Core Team, 2015). A one-way ANOVA followed by a pairwise *t*-test was used to explore variations in OTU richness and Shannon’s diversity levels across the sea regions and habitat types.

Principal coordinate analysis (PCoA) was used to visualize dissimilarities among the fungal communities across the sea regions and habitat types based on Hellinger-transformed Bray–Curtis distances. Prior to our PCoA analyses, OTUs represented by fewer than 5 reads were removed, as rare OTUs have a marginal effect on subsequent multivariate statistical analyses (Lindahl et al., 2013; Tedersoo et al., 2014). Raw read counts were then normalized using the package DESeq2 in R (Senés-Guerrero and Schüßler, 2016) and relative abundance of matrix was obtained for calculation of Bray–Curtis distances. The influences of latitude, sea region and habitat type on the difference of fungal beta-diversity were explored with adonis functions with permutations = 200. Pearson’s correlation coefficient, the 95% confidence interval on regressions, and *P*-values were calculated to explore the associations between OTU richness and latitude, geographical distance and Bray–Curtis distance, using lm function followed by an ANOVA test in the vegan package.

## Results

**FIGURE 2:**
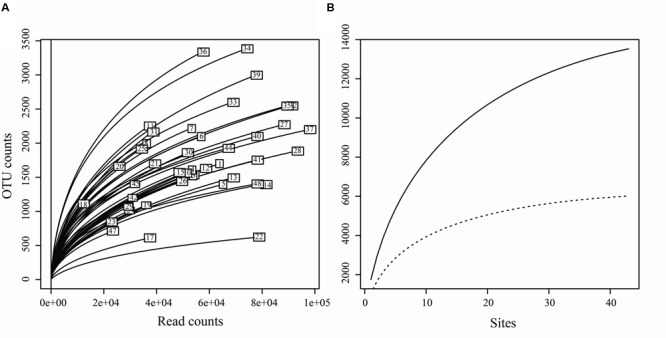
**(A)** Rarefaction curves for each sample, relating the number of OTUs detected depending on the sequencing effort. **(B)** Accumulation curves of total OTUs (solid line) and fungal OTUs (dotted line) detected to the number of the sampling sites.

Of the 3,250,710 raw sequences, 2,396,219 sequences were retained after the filtering and denoising processes, and subsequently clustered into 23,115 OTUs. After singleton removal (41.47% of OTUs), 13,530 OTUs were retained with an average of 55,503 sequences per sample (range: 12,404 – 97,692). Among them, 6,013 OTUs belonged to the fungal kingdom, accounting for 37.76 % (901,236 sequences) of the total sequences (**Supplementary Table S3**). Other OTUs belong to Animalia (0.0002% of sequences), Plant (0.25%) and Protista (0.34%). In addition, 54.10% of OTUs (61.65% of sequences) failed to be assigned to any known kingdom based on current database (**Supplementary Figure S1**). The rarefaction curves relating to the OTUs detected, depending on the sequencing effort, showed that most samples did not approach an asymptote (except perhaps sites 17, 14, 22, and 48) (**Figure 2A**). Furthermore, the total OTUs accumulation curve did not approach an asymptote (**Figure 2B**).

### Taxonomic Assignment and Compositions of Fungal Community

For the fungal kingdom, the phylum-level assignment of the 913 OTUs remained elusive, and the other 5,100 OTUs spanned six phyla, 23 known classes, 84 orders and 526 genera (**Supplementary Table S3**). From these, 72.08% of OTUs (82.85% of fungal sequences) were assigned to the Ascomycota, followed by Basidiomycota (8.20% of fungal OTUs, 3.42% of fungal sequences). Chytridiomycota, Glomeromycota, Rozellomycota and Zygomycota were recovered with a small proportion (4.54% of OTUs, 1.75% of sequences) (**Supplementary Figure S1**). The OTUs accumulation curve for the fungal data set (before reads resampling) nearly approached an asymptote (Chao1 = 6,266, **Figure 2B**), indicating that our data could basically reflect the fungal biodiversity in the Chinese intertidal sediments.

In Ascomycota, Sordariomycetes (22.93% of fungal OTUs, 22.08% of fungal sequences) was found to be the most abundant, followed by Dothideomycetes (18.94%, 21.75%), Eurotiomycetes (16.15%, 18.53%) and Leotiomycetes (5.24%, 2.58%) (**Supplementary Table S4**). For the majority of sampled sites, these classes appeared to be the dominant colonizers (**Figure 1B**). Within Basidiomycota, Agricomycetes (5.84%, 1.67%) was the most abundant class, followed by Tremellomycetes (1.20%, 0.74%) and Microbotryomycetes (0.58%, 0.42%).

There were 3,175 OTUs (67.28% of fungal sequences) that could be assigned to 526 fungal genera (**Supplementary Table S5**). Among them, 23 genera were present in all of the samples. *Penicillium* (329 OTUs) appeared with the most relative abundance of sequences (10.48%), followed by *Pseudeurotium* (18 OTUs, 4.59%), *Phaeosphaeria* (68 OTUs, 4.08%), *Aspergillus* (143, 3.52%), *Talaromyces* (104 OTUs, 2.55%), *Gibberella* (11 OTUs, 2.22%) and *Cladosporium* (21 OTUs, 2.17%). The third most abundant genus *Taifanglania* (14 OTUs, 4.57%) appeared in 97.67% of the samples. Fifty-three genera with a very small proportion of reads (0.10%) were detected only in a single sample.

The 20 most abundant OTUs were listed in **Table 1**. According to our strict criterion applied for the classification of OTUs at species level, 99 species could be identified from 101 RS, including 71 species in Ascomycota, 21 in Basidiomycota and 7 in Zygomycota (**Supplementary Table S6**). Based on relative read abundance, the five most abundant species (from highest to lowest) were *Pleurostomophora richardsiae, Malassezia restricta, Aspergillus tritici, Bionectria epichloe*, and *Exophiala xenobiotic*. Moreover, eight species, *Pleurostomophora richardsiae, Bionectria epichloe, Cladorrhinum bulbillosum, Aspergillus tritici, Lophiostoma helminthicola, Exophiala xenobiotic, Trichurus dendrocephalus*, and *Mucor circinelloides*, were present in more than half of all samples.

**Table 1:** List of the 20 most abundant of OTUs across the Chinese intertidal zone.

OTU number	Occurrence	Read counts	Reference sequence	Taxonomy of reference sequence	Similarity (%)	Coverage (%)
OTU_4986	30	42,318	GU316272	Uncultured fungus	88.89	86
OTU_17787	43	32,942	KJ812279	*Phaeosphaeria spartinae*	100	96
OTU_7476	29	30,307	FJ553511	Uncultured Agaricomycetes	95.02	94
OTU_18051	43	28,494	HM589327	Ascomycota sp.	97.42	98
OTU_13157	23	22,166	KC832906	*Peziza domiciliana*	92.12	90
OTU_11126	32	20,566	HQ876044	*Scheffersomyces spartinae*	98.44	100
OTU_13183	43	19,880	KM386990	*Penicillium concentricum*	99.45	100
OTU_17074	43	17,320	JX496088	*Paraconiothyrium cyclothyrioides*	98.16	100
OTU_17644	38	17,320	KJ026971	*Taifanglania* sp.	92.19	79
OTU_16100	43	16,544	KJ996101	*Fusarium equiseti*	99.4	100
OTU_11859	28	16,534	KJ775700	*Talaromyces diversus*	97.87	100
OTU_14982	41	15,337	KC478608	*Alternaria alternata*	100	100
OTU_18026	17	14,560	KJ026971	*Taifanglania* sp.	92.86	79
OTU_16697	43	13,115	KM999223	*Cladosporium cladosporioides*	98.79	100
OTU_6071	24	12,321	FJ553511	Uncultured Agaricomycetes	89.64	100
OTU_13984	43	12,074	KJ939431	*Penicillium oxalicum*	100	100
OTU_16575	43	11,329	KJ599668	*Talaromyces amestolkiae*	92.12	100
OTU_17750	43	9,884	KM066188	*Talaromyces macrosporus*	99.37	100
OTU_16259	43	8,686	FJ235981	Fungal sp.	100	100
OTU_17273	35	7,604	KF060220	Uncultured Pleosporales	92.76	93

### Comparison of α-Diversity among Sea Regions and Habitat Types

**FIGURE 3:**
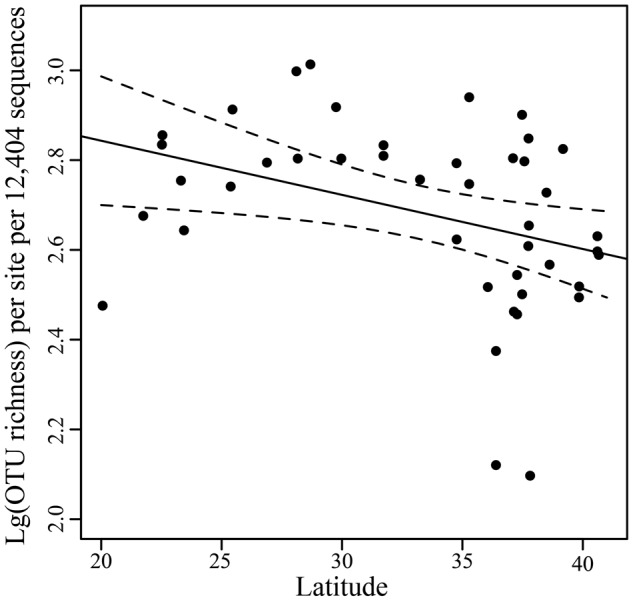
**Scatterplots of OTU richness vs. latitude.** Linear regression line and the 95% confidence limits (dashed) are shown.

After normalization of sequences by resampling 12,404 reads, comparative analyses of fungal community diversity across different sites were performed. OTU richness observed ranged from 132 to 1,031 per sample with a mean (±SD) value of 529 ± 215 (**Supplementary Table S1**). The correlation between OTU richness and latitude was weakly significant (*r* = -0.3614, *P* = 0.0173) (**Figure 3**).

**FIGURE 4:**
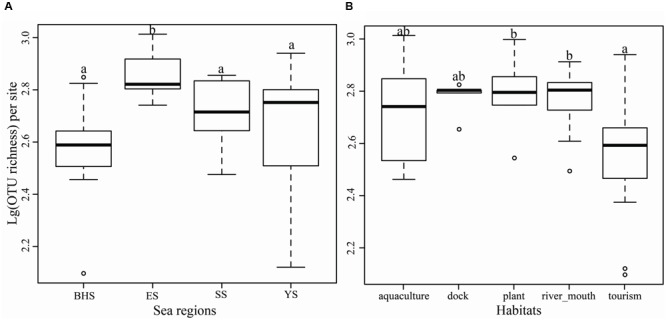
**Operational taxonomic units richness per site in four sea regions (A)** and five types of habitat **(B)** as demonstrated by boxplot with median and 95% confidence intervals displayed. Bars without shared letters indicate significant differences at the level of *P*-value = 0.05.

Significance in OTU richness between four sea regions was found by a one-way ANOVA analysis (*F* = 5.347, *P* = 0.0035), and the ES was found to have a higher level of OTU richness (744 ± 166) than the other three seas (*P* < 0.05) (**Figure 4A**). The five habitat types were found to be significantly different in OTU richness (*F* = 2.975, *P* = 0.0312). The two types, river mouth habitat and plant rich habitat, harbored higher relative levels of OTU richness than the type of tourism (**Figure 4B**). Surprisingly, neither sea regions (*F* = 2.154, *P* = 0.1090) nor habitat types (*F* = 0.908, *P* = 0.4690) differed from each other in Shannon indices (**Supplementary Figure S2**).

The distribution of 19 major taxonomic groups (mainly at class level) in terms of relative abundance is shown in **Figure 1B**. One-way ANOVA tests revealed that all of these major taxonomic groups were found to be not significantly different in relative abundance among four sea regions (*P* > 0.05). A similar observation occurred in the five types of habitat, with the exception of the docks area where the level of relative abundance of Agaricomycetes in sediments was obviously higher than in other habitat types (*F* = 3.997, *P* = 0.0084).

### β-Diversity and the Effect of Variables

**FIGURE 5:**
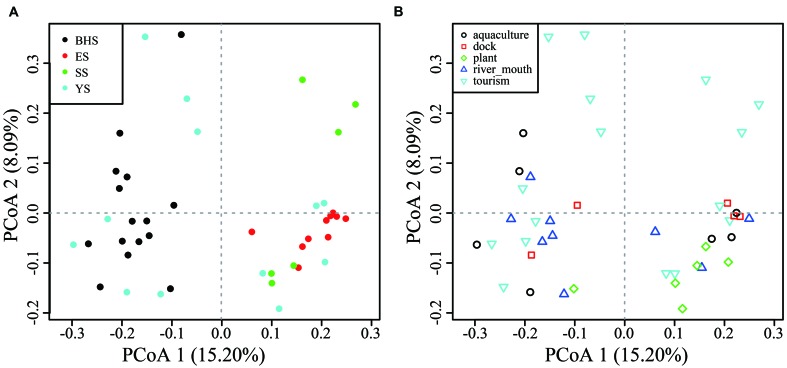
**Principal coordinate analysis (PCoA) plots of (A)** four Chinese seas and **(B)** five types of habitat on the basis of Hellinger-transformed Bray–Curtis distance.

Among fungal OTUs, only 1,422 OTUs were present in all sea regions and 1,529 in all habitat types (**Supplementary Figure S3**), indicating a notable variability of species compositions across the intertidal zone. On the basis of PCoA plots, obvious distinctions in community compositions were found between the BHS and ES, BHS and SS, but not among the YS, ES and SS (**Figure 5A**). All habitat types were not found to obviously differ from each other in their community compositions (**Figure 5B**).

**FIGURE 6:**
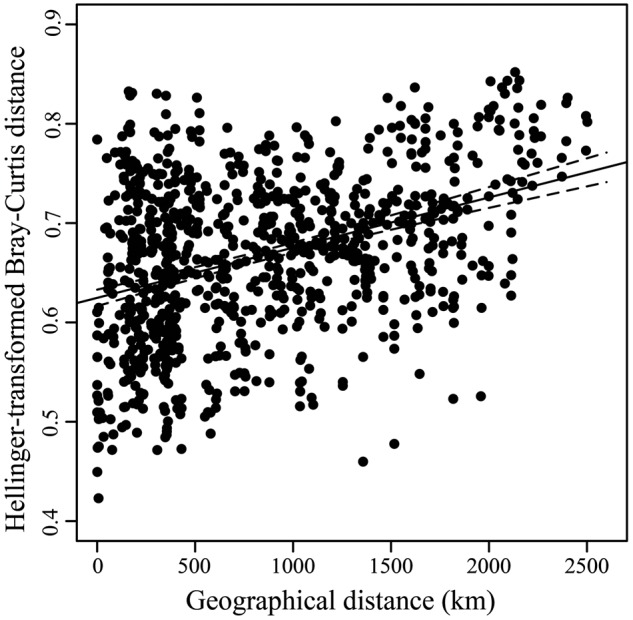
**Scatterplots of Hellinger-transformed Bray-Curtis distance vs. geographical distance.** Linear regression line and the 95% confidence limits (dashed) are shown.

ADONIS tests revealed that latitude and sea region significantly influenced the fungal beta-diversity in all samples (*P* = 0.001), whereas the influence of habitat type was not significant (*P* = 0.068) (**Table 2**). However, a significant influence (*P* = 0.001) was found when habitat type was further separated by different sea regions. Geographical distance was found to be significantly related to the structure of the fungal community, based on a Pearson correlation analysis (*r* = 0.3834, *P* < 0.001), indicating sediments collected from intertidal regions close together in space tended to have similar taxa present (**Figure 6**).

**Table 2:** ADONIS analysis based on Hellinger-transformed Bray–Curtis distance.

Environmental variable	*R*^2^	*P*
Latitude	0.111	0.001^∗^
Sea region	0.1711	0.001^∗^
Habitat type	0.1135	0.066
Habitat type separated by sea regions	0.4724	0.001^∗^

*The asterisk indicates significance at the level of *P-*value = 0.05.*

## Discussion

### Accuracy of the Classification Method Used

For classification of the entire ITS and partial ITS sequences, BLASTn is a reliable method that has been reported to be consistently better than MEGAN and SAP (Porter and Golding, 2011). NBC is an ideal alternative classification method for environmental rRNA regions such as the ITS that cannot be aligned with confidence (Wang et al., 2007; Deshpande et al., 2016). Particularly with the use of a 50% bootstrap cut-off, NBC showed higher classification accuracies at the genus level compared to BLASTn (Porras-Alfaro et al., 2014). For classification accuracy, in this study, the two methods of BLASTn and NBC (with 50% bootstrap cut-off) were applied to assign a RS into a certain taxonomic position.

According to our results, 514 genera (50.22% of total OTUs) and 448 genera (50.67% of total OTUs) were annotated using BLASTn and NBC-based methods (**Table 3**), respectively, which indicates more taxa at the genus level can be identified using BLASTn than by NBC. Among these OTUs, 67.12% of OTUs were assigned into the same genus by both methods, supporting previous findings that there is a significant congruency between the two taxonomies (Porras-Alfaro et al., 2014). On higher taxonomic levels, higher congruencies were found at the level of family (72.23%), order (83.96%) and class (87.77%). These data indicate our standard method applied for OTUs annotation can provide an accurate and robust classification for ITS2 sequences recovered from our sediment samples.

**Table 3:** Comparison of OTU number annotated by BLASTn and NBC at different levels of taxonomy.

	Class	Order	Family	Genus
Blastn	4,507	4,172	3,230	3,020
NBC	4,333	3,855	2,944	3,047
Total OTU counts of annotation available based on two methods	4,651	4,309	3,435	3,519
Consistent percent of OTUs (%)^∗^	87.77	83.96	72.23	67.12

*The asterisk indicates that percent of convergence is calculated with the number of OTUs that have the same annotation by the two methods, divided by the total number of OTUs annotated by the two methods.*

### Taxonomic Coverage With Diverse Fungal Groups

To our knowledge, this is the first investigation of fungal diversity in the Chinese intertidal zone of such a large scale using molecular tools. Diverse taxonomic groups spanning six fungal phyla had been detected in sediment of our sampled regions, indicating the intertidal region is an important habitat for the colonization of fungi (Kohlmeyer and Kohlmeyer, 1979; González et al., 1998). No Microsporidia sequences were found in our samples, which might be due to considerable mismatching with this group by primers of ITS3-ITS4 (Tedersoo et al., 2015). More than half of all RS (54.10%) recovered poorly matched the available database and failed to be assigned to any known taxa, which can probably be attributed to the paucity of reference sequences currently available in the database (Kõljalg et al., 2013; Nilsson et al., 2015). This also perhaps suggests that there is a considerable undescribed fungal population that is yet to be recognized.

Jones et al. (2015) reviewed the marine fungi revealed through traditional approaches and concluded that Halosphaeriaceae is the largest family with 141 species in 59 genera. In this study, only a small proportion (0.09%) of environmental sequences affiliated to Halosphaeriaceae were detected from 32 samples, and some were classified into five genera (i.e., *Ceriosporopsis, Corollospora, Lignincola, Remispora, Sigmoidea*). Most taxa in this family are saprobic in marine habitats, and few are transitional species found in freshwater and brackish water habitats (Sakayaroj et al., 2011). Thus, fungal genera/species affiliated to Halosphaeriaceae are usually considered as obligate marine fungi (Jones et al., 2006). For example, the OTU_13192 composed of 62 reads was identified as *Corollospora maritima*. Species of *Corollospora* are typical arenicolous fungi which are morphological adapted to the intertidal habitat with thick, hard and carbonaceous ascocarp walls (González and Richard, 2010). *Corollospora* species have known functions of decomposers of dead wood, leaves or seaweed (Kohlmeyer and Kohlmeyer, 1979).

Among the genera uncovered in this study, most are known as terrestrial fungi and more than one fifth are obligate pathogens of various plants (**Supplementary Table S5**). Within Basidiomycota, the majority of recovered sequences were assigned to Agaricomycetes, particularly Polyporales and Agaricales. This finding is surprising, as many species of the two orders are typically terrestrial wood decomposers (Hiscox et al., 2015) and do not live in marine sediments. Therefore, it is speculated that most fungi recovered from the ocean may be derived from terrestrial environments via rivers or terrestrial runoff (González and Richard, 2010; Li et al., 2016). On the other hand, some members of Agaricales (e.g., *Cortinarius, Lepiota*) have previously been obtained from mangrove sediments, suggesting a potential function as decomposers of dead wood or leaf litter (Jones et al., 2006). The intertidal region is physically dynamic with extreme shifts in temperature, salinity, sunlight radiation, water content and intermittent nutritional substrate availability (González and Richard, 2010). It raises the question of the adaptation of these members to environmental conditions and of the roles they play in the organic matter decomposition process.

Filamentous fungal forms (such as Sordariomycetes and Dothideomycetes in Ascomycota, and Agricomycetes in Basidiomycota) were found to be the dominant taxonomic groups in this study, which differs from previous findings that the marine Dikarya is dominated by phylotypes capable of living as yeasts in water columns of the deep sea (Bass et al., 2007) and European coast (Richards et al., 2015). A possible explanation is that filamentous fungi are preferentially suited for solid substrates rich in organic matter such as soils and sediments (Richards et al., 2012). Among 130 genera revealed from subtidal sediments of the BHS and YS by our previous study (Li et al., 2016), 103 genera can be found in this study. This raises an argument that the majority of fungi in the benthic sediment of coastal ocean could have their phylogenic origins in intertidal sediment. Moreover, some ascomycetes yeasts (i.e., *Debaryomyces, Candida, Pichia* and *Saccharomyces*) and basidiomycetes yeasts (i.e., *Cryptococcus, Malassezia, Rhodosporidium, Rhodotorula* and *Sporobolomyce*s) revealed in our study were also detected from costal sediments of European oceans (Richards et al., 2015) and deep sea (Lai et al., 2007; Edgcomb et al., 2011). These observations encourage us to speculate that the intertidal sediment perhaps represents a “seed bank” for fungi inhabiting marine habitats of the Chinese seas.

Relatively lower levels of the fungal basal lineages were recovered, which include six genera in Chytridiomycota (i.e., *Olpidium, Phlyctochytrium, Powellomyces, Rhizophlyctis, Rhizophydium* and *Spizellomyces*) and nine in Zygomycota (i.e., *Actinomucor, Choanephora, Gongronella, Lichtheimia, Mortierella, Mucor, Pilobolus, Rhizopus*, and *Umbelopsis*). Considering bias of the PCR processes and ITS primers toward Dikarya fungi (Richards et al., 2012; Ishii et al., 2015; Tedersoo et al., 2015), phylotypes of the two phyla may be more diverse and numerous than we recovered here. Many members of these “lower fungi” had been found to be decomposers of pollen, leaves and wood in marine ecosystems (Phuphumirat et al., 2016) or pathogens prevalent in marine algae, animals and even human (Scholz et al., 2016). For example, RS of OTU_7032 has a value of 100% identity and coverage against the sequence of *Actinomucor elegans* (GenBank accession number: JN205825) in the UNITE Community^1^ and this fungus was reported to be an emerging fungal pathogen capable of causing invasive mucormycosis in humans (Mahmud et al., 2012).

Interestingly, five mycorrhizal genera (i.e., *Ambispora, Claroideoglomus, Diversispora, Funneliformis* and *Glomus*) were detected in more than half of all samples. Moreover, RS of OTU_2675 had a value of 100% identity and coverage against uncultured Glomeraceae sp. (KC965737), which was recovered from soil of zonal patterned-ground ecosystems across the North American Arctic (Timling et al., 2014). Mycorrihizal fungi are believed to be terrestrial or important in oligotrophic waters by supplying aquatic plants with solid-phase bound nutrients (Baar et al., 2011). The only report of the occurrence of Glomeromycota in the ocean was by Orsi et al. (2013) who detected two genera (*Glomus* and *Diversispora*) from sediment samples from the Benguela Upwelling System. Further investigation is required on their adaptation and survival strategies in the ocean, which is critical to understand their ecological function.

Members of Rozellomycota are known as endoparasites of other chytrids, oomycetes, amoebae and algae (Manohar and Raghukumar, 2013; Ishii et al., 2015). For example, two endonuclear parasites identified as *Paramicrosporidium* were recently recognized from free-living naked amoebae (Corsaro et al., 2014). In this study, 121 OTUs were assigned to this phylum, which were from 22 samples spanning all of the sea regions. This is consistent with previous data suggesting this phylum is widespread in marine environments (Richards et al., 2012, 2015; Manohar and Raghukumar, 2013). RS of OTU_11336 has a value of 100% identity and coverage against the sequence of one uncultured soil fungus identified as Rozellomycota sp. (FJ197921) in the UNITE Community. Another two RS of OTU_11128 and OTU_13176 matched against uncultured Rozellomycota sp. (JQ666560 and JQ666534) with 100% identity and coverage, respectively, which were recovered from forest soil of Changbai Mountain of China (Han et al., 2016). Additionally, 59 OTUs that had less than 95% identity against sequences in the database, indicating a substantial diversity of Rozellomycota sequences that represent undescribed and diverse taxonomic groups inhabiting in the sediment of the Chinese intertidal region.

### Potential Factors Influencing Spatial Variation of Fungal Communities

To date, the number of marine fungi reported from tropical regions is more than from other regions (Jones, 2000, 2011). The weakly significant correlation between OTU number and latitude may present a latitudinal diversity gradient in fungal richness, in agreement with a previous study (Liu, 2013). The prominent characteristic of fungal communities across our sampled regions is that the ES significantly differed from the other three sea regions in compositions of the fungal community, with a more compact ordination in a PCoA plot (**Figure 5A**). Additionally, the level of OTU richness in the ES is significantly higher than that of the YS (with higher latitude than the ES) and SS (with lower latitude) (**Figure 4**). These findings may be explained partially by the huge discharge of the Yangtze River with about 4.7–5 × 10^8^ tons of sediment (carrying terrigenous organic matters) per year into the local ocean. Under the action of the East China Sea Coastal Current, these sediments are then pushed to the coasts of the East Sea forming the offshore mud patches (Song, 2009) which likely favor organism growth. Moreover, the strong influence of the Yangtze River will probably result in a closer affinity of fungal communities in the ES than other sea regions.

Mangrove leaf litter and dead wood provide an ideal habitat for marine fungi (Jones, 2000; Jones et al., 2006; Phuphumirat et al., 2016). With 454-pyrosequencing technology using ITS1 and ITS2 barcodes, Arfi et al. (2012) found that Agaricomycetes is the dominant class in the anoxic mangrove sediments of Saint Vincent Bay. Whereas Sordariomycetes (relative abundance is 47.47%) and Dothideomycetes (24.52%) are the dominant classes in our mangrove samples, indicating that fungal diversity may vary from one mangrove to another (Jones, 2000). Surprisingly, our data suggested no significances in fungal communities between plant habitat and other habitat types (**Figure 5B**). Although of this, there are 24 genera (e.g., *Cortinarius, Lepiota, Leptodontidium, Pochonia*, and *Sistotrema*) detected only in mangrove sediments, suggesting a special community (Jones et al., 2006; Pang et al., 2011). Interestingly, high relative abundance (40.12%) of Rozellomycota was detected in site 14 which is covered by the reed *Phragmites australis*.

Coastal and estuarine ecosystems in China are suffering intense disturbance from human activities, such as the input of nutrients, sewage, pesticides and industrial wastes (He et al., 2014). These human activities profoundly disturb both freshwater and marine fungal communities (Tsui et al., 1998; González and Richard, 2010). Therefore, it is not surprising that some common fungi were predominant in their relative abundance in most aquaculture sites. For example, high relative abundances of *Penicillium* (52.54%) and *Aspergillus* (15.35%) were found in site 37. Other aquaculture sites, including sites 20, 7, 15, and 40, exhibited high relative abundance of *Gibberella* (60.01%), *Cladosporium* (11.14%), *Cladosporium* (11.62%) and *Penicillium* (13.24%), respectively. This is consistent with a previous finding that *Penicillium, Aspergillus, Trichoderma* and *Cladosporium* were obtained by culture methods with clear predominance from marine shellfish farming areas along the western coast of France (Sallenave-Namont et al., 2000). A similar observation occurred in some sites located in dock, tourism and river mouth areas, where with predominant occurrence was of the above fungal genera, as well as *Pseudeurotium, Phaerosphaeria, Taifanglania*, and *Talaromyces*.

On the other hand, a PCoA plot showed that no significant difference in fungal communities was detected among sediments collected from tourism, dock, aquaculture and river mouth areas, which are subjected to distinct anthropogenic influences (Nordlund et al., 2014; Whitfield and Pattrick, 2015). Nutritional conditions, temperature, pH, salinity, and other characteristics of marine sediment have been found to have a strong effect on the composition of the fungal community (Jones, 2000; Guo et al., 2015; Li et al., 2016; Tisthammer et al., 2016; etc.). Therefore, we speculated that the difference in physicochemical properties of sediments collected from different sites with large geographical origin might mask an effect of habitat types.

To conclude, our study showed that the Chinese intertidal region harbors a high-level fungal diversity with an obvious spatial variation of community composition. Such a high species richness, coupled with the fact that many well-known decomposer representatives were highlighted, suggests the important ecological roles of fungi in intertidal regions. However, we should aware that the metabarcoding-based approach for estimation of fungal diversity suffers from certain limitations mainly because of paucity of reference sequence of little-studied phyla (e.g., Chytridiomycota, Rozellomycota) in public database (Ishii et al., 2015) and methodological bias (e.g., bias of primer and annotation, varying PCR efficiency in different samples) (Richards et al., 2012; Lindahl et al., 2013; Tedersoo et al., 2015). Finally, to get insights into the activity of marine fungi in such habitat and their associated functions, complementary rRNA and mRNA approaches will be necessary in future studies.

## Author Contributions

WL: Conceived and designed the work that led to the submission, programmed most of the bioinformatic analysis, analyzed data, drafted most part of the manuscript and approved the final version of our manuscript. MW: Processed part of the raw data, programmed part of the bioinformatic analysis, submitted the raw data to SRA and submitted our manuscript to *Frontier in Microbiology*. XB: Collected the sediment samples. JG: Programmed part of the bioinformatic analysis. LC: Drafted part of the manuscript and revised our manuscript.

## Conflict of Interest Statement

The authors declare that the research was conducted in the absence of any commercial or financial relationships that could be construed as a potential conflict of interest.

## Footnotes

1 https://unite.ut.ee

## Supplementary Material

FIGURE S1 **Taxonomic groups (mainly at the class level) of (A)** 6,210 OTUs and **(B)** 915,222 reads across our sampling sites.

FIGURE S2 **Shannon index of four sea regions (A)** and five types of habitat **(B)** as demonstrated by boxplot with median and 95% confidence intervals displayed. Bars without shared letters indicate significant differences at the level of *P*-value = 0.05.

FIGURE S3 **Venn diagrams of OTU compositions among four sea regions (A)** and five types of habitat **(B)**.

TABLE S1 **Detailed information of 43 sampling sites in the forms of longitude-latitude and α-diversity after read resampling**.

TABLE S2 **Primer identifier barcodes of 43 sampling sites**.

TABLE S3 **OTU annotation and distribution of read counts across our sampling sites**.

TABLE S4 **Abundance distribution of OTUs at the levels of phylum, class and order**.

TABLE S5 **Information of 526 genera identified including counts of OTU and read, distribution in 43 sites, sea regions and habitat types**.

TABLE S6 **Abundance distribution of 99 taxa at the level of species**.

TABLE S7 **Nucleotide of representative sequences**.
