# Supplementary figures and images for: A High-Level Fungal Diversity in the Intertidal Sediment of Chinese Seas Presents the Spatial Variation of Community Composition
Source: Front Microbiol. 2016 Dec 23;7:2098. doi: 10.3389/fmicb.2016.02098 (PMC5179519; doi:10.3389/fmicb.2016.02098)

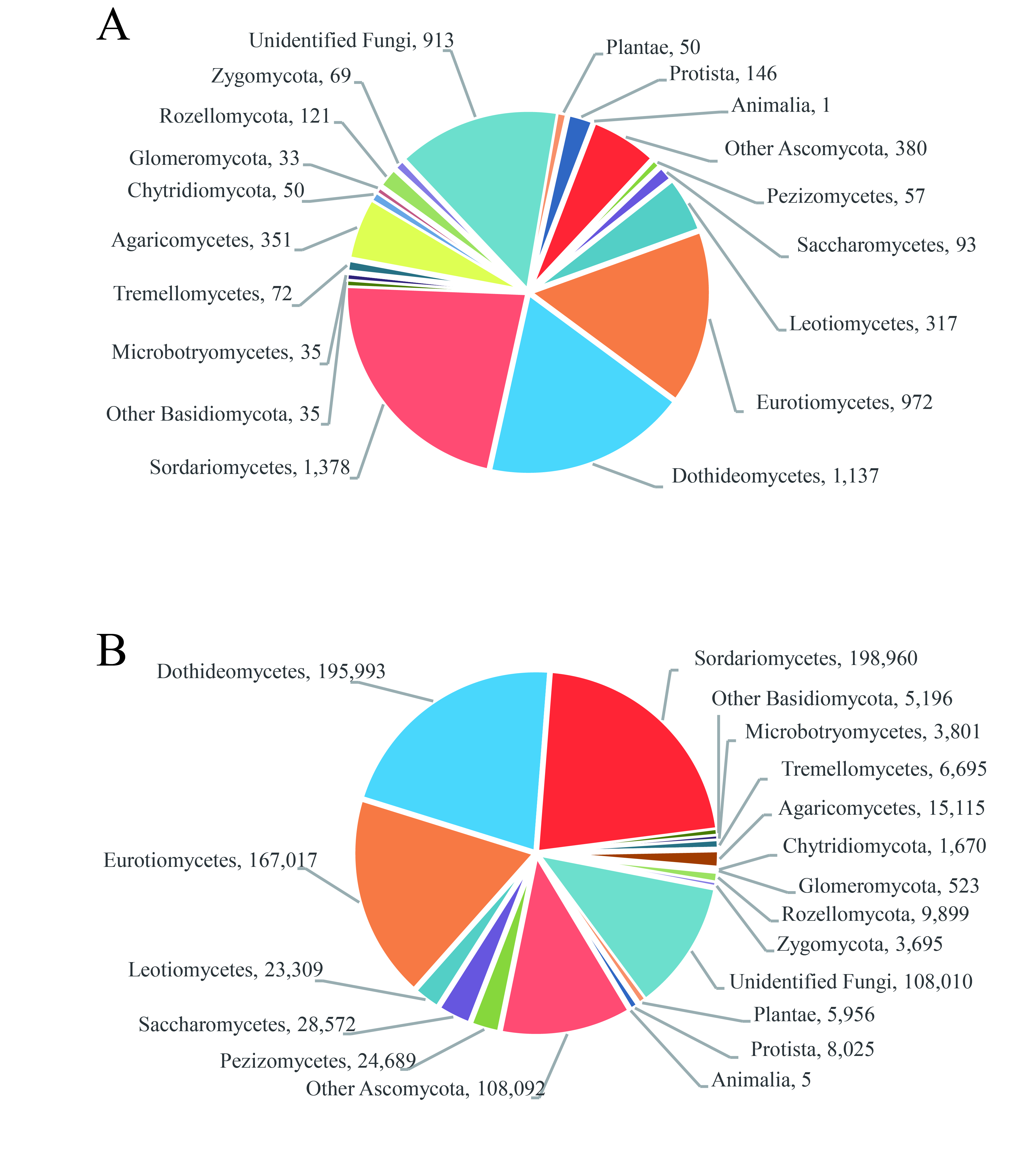

Supplement: FIGURE S1 — Taxonomic groups (mainly at the class level) of (A) 6,210 OTUs and (B) 915,222 reads across our sampling sites. [file Image_1.JPEG]

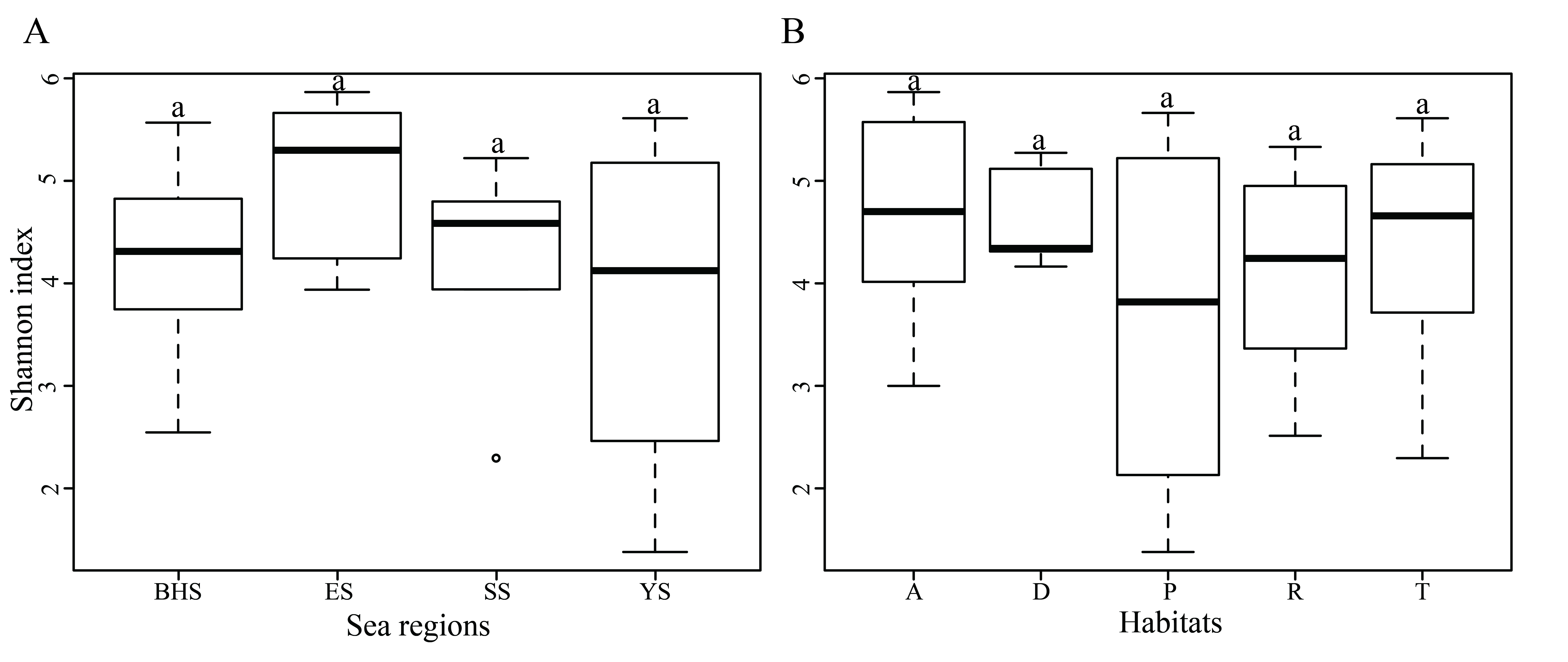

Supplement: FIGURE S2 — Shannon index of four sea regions (A) and five types of habitat (B) as demonstrated by boxplot with median and 95% confidence intervals displayed. Bars without shared letters indicate significant differences at the level of P-value = 0.05. [file Image_2.JPEG]

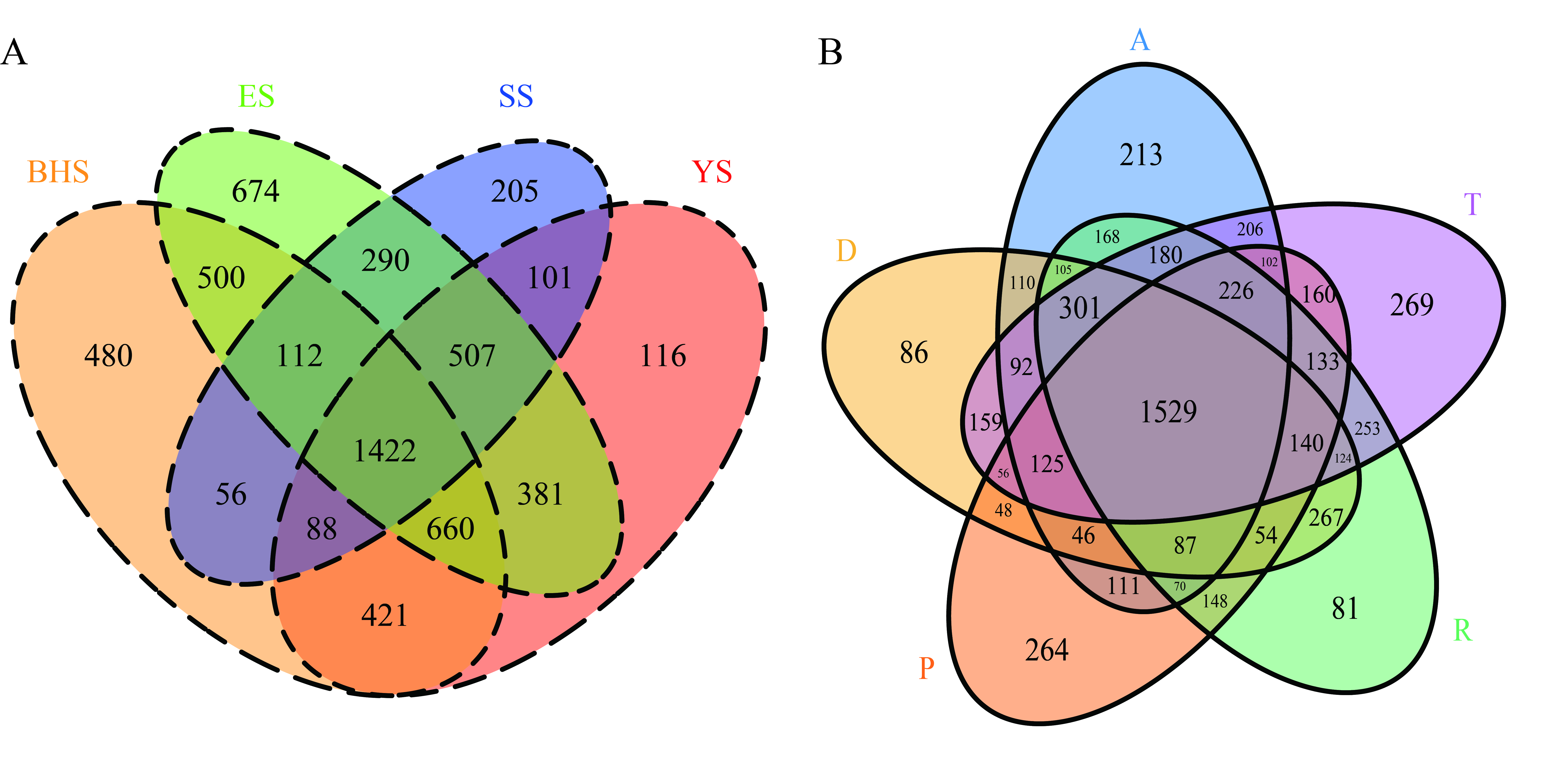

Supplement: FIGURE S3 — Venn diagrams of OTU compositions among four sea regions (A) and five types of habitat (B). [file Image_3.JPEG]
